# Epigenetic silencing of the *ANKRD26* gene correlates to the pro-inflammatory profile and increased cardio-metabolic risk factors in human obesity

**DOI:** 10.1186/s13148-019-0768-0

**Published:** 2019-12-04

**Authors:** Antonella Desiderio, Michele Longo, Luca Parrillo, Michele Campitelli, Giuseppe Cacace, Sonia de Simone, Rosa Spinelli, Federica Zatterale, Serena Cabaro, Pasquale Dolce, Pietro Formisano, Marco Milone, Claudia Miele, Francesco Beguinot, Gregory A. Raciti

**Affiliations:** 1 URT Genomics of Diabetes, Institute of Experimental Endocrinology and Oncology, National Research Council, Via Pansini 5, 80131 Naples, Italy; 2 Department of Translational Medicine, Federico II University of Naples, Via Pansini 5, 80131 Naples, Italy; 3 Department of Public Health, Federico II University of Naples, Via Pansini 5, 80131 Naples, Italy; 4 Department of Clinical Medicine and Surgery, Federico II University of Naples, Via Pansini 5, 80131 Naples, Italy

**Keywords:** *ANKRD26*, Obesity, Epigenetic silencing, DNA methylation, Cardio-metabolic risk factors

## Abstract

**Background:**

Obesity is a major worldwide threat to human health. Increasing evidence indicates that epigenetic modifications have a major impact on the natural history of this disorder. *Ankyrin Repeat Domain 26* (*Ankrd26*) is involved in the development of both obesity and diabetes in mice and is modulated by environmentally induced epigenetic modifications. This study aims at investigating whether impaired *ANKRD26* gene expression and methylation occur in human obesity and whether they correlate to the phenotype of these subjects.

**Results:**

We found that downregulation of *ANKRD26* mRNA and hyper-methylation of a specific region of the *ANKRD26* promoter, embedding the CpG dinucleotides − 689, − 659, and − 651 bp, occur in peripheral blood leukocytes from obese compared with the lean subjects. *ANKRD26* gene expression correlates inversely to the percentage of DNA methylation at these 3 CpG sites. Luciferase assays reveal a cause-effect relationship between DNA methylation at the 3 CpG sites and *ANKRD26* gene expression. Finally, both *ANKRD26* mRNA levels and CpG methylation correlate to body mass index and to the pro-inflammatory status and the increased cardio-metabolic risk factors of these same subjects.

**Conclusion:**

Downregulation of the *ANKRD26* gene and hyper-methylation at specific CpGs of its promoter are common abnormalities in obese patients. These changes correlate to the pro-inflammatory profile and the cardio-metabolic risk factors of the obese individuals, indicating that, in humans, they mark adverse health outcomes.

## Background

Obesity is a chronic disorder associated with high risks of adverse health outcomes [1]. It is also a major risk factor for several metabolic diseases, including impaired glucose tolerance, type 2 diabetes (T2D), dyslipidemia, hypertension, and cardiovascular diseases (CVDs) [2]. Because of its globally increasing prevalence, obesity is one of the main clinical challenge in the twenty-first century [1, 3]. However, the mechanistic factors leading to obesity remain, in most cases, unclear [4].

Current evidence indicates that the recent rise in obesity prevalence is determined by unhealthy gene-environment interactions [5, 6]. Several studies have also documented that environmental hits may cause epigenetic changes, which, in turn, alter gene function and affect disease susceptibility [7, 8]. Epigenetics has been proposed to link genes and environment and may contribute to the worldwide increase in obesity prevalence. Very recent findings, both in animal models and in humans, have also documented the relationship between the risk of obesity and the epigenetic impairment of gene function, which are closely associated with weight gain and metabolic traits [4, 9–14]. For instance, in a candidate gene study for the *preproopiomelanocortin* locus, Kuehen et al. have identified an epigenetic variant of CpG methylation that is associated with the individual risk for obesity [12]. Furthermore, Pfeiffer et al. have demonstrated that the gene expression and DNA methylation of their candidate gene, the *hypoxia-inducible factor 3A*, is related to adipose tissue dysfunction, making this gene an important factor involved in the etiology of obesity [13]. However, whether these associations are causal and the direction of the causations remains controversial. Some studies indeed suggest that alterations in DNA methylation are predominantly the result of increased body mass index (BMI)/adiposity typical of obesity [14, 15]. In an association study of BMI and differential methylation, Mendelson et al. have demonstrated that among the 83 novel differentially methylated CpGs related to BMI, the alterated methylation at one only specific CpG site has a causal effect on BMI and adiposity-related traits, while other 16 CpGs were secondary to differences in BMI [14]. In a further association study, Wahl et al. have also reported that the changes in DNA methylation at the majority of the identified 187 CpG sites are consequences and not the cause of adiposity [15]. Therefore, whether epigenetic changes affect obesity development or *vice versa* still represents an open question and will need to be assessed on a case-by-case basis.

Through a methylated DNA immuno-precipitation sequencing (MeDIP-seq) approach, we have recently demonstrated that high-fat feeding triggers a massive DNA methylation reprogramming in genes involved in developmental, metabolic, and transcriptional processes in mice [16]. In addition, our recently published findings in the mouse model have identified *Ankrd26* as a gene undergoing epigenetic changes in response to high-fat feeding [17]. High-fat exposure causes a specific hyper-methylation of the *Ankrd26* promoter at the − 436 and − 431 bp CpG sites, which is dependent upon enhanced binding of the de novo DNA methyltransferases 3a and 3b to the same *Ankrd26* promoter region. These changes are followed by downregulation of *Ankrd26* expression in the white fat depots [17]. *ANKRD26* is located at chromosome 10p12, a locus previously associated with maternally inherited obesity in humans [18]. Only two rare single-nucleotide polymorphisms (SNPs) at the *ANKRD26* gene, the rs139049098 (minor allele frequency, MAF: *C* = 0.0004/2) and the rs191015656 (MAF: *A* = 0.0004/2), have been so far associated with severe obesity in humans [19]. Very recently, the *ANKRD26* gene SNP, rs7081476 (MAF: *C* = 0.0541/271), has been associated across multiple studies to a variety of diseases, including diabetes and CVDs, and to different endophenotypes, such as BMI, high-density lipoprotein cholesterol (HDL-C), triglycerides (TG), blood glucose, and hemoglobin A1c (HbA1c) [20]. In addition to ours, other groups have further described *Ankrd26* as a gene involved in the regulation of feeding behavior and in the development of both obesity and diabetes in mice [21–23]. Mutant mice with a partial inactivation of this gene (*Ankrd26* MT) show obese and diabetic phenotypes which result from marked hyperphagia rather than reduction in energy expenditure or activity [21, 22]. Indeed, the C-terminal deletion of this gene, in vivo, in the *Ankrd26* MT mice, leads to defects in primary cilia formation in central nervous system areas which regulate both food intake and energy homeostasis [23].

Altogether, this evidence suggests that the *Ankrd26* gene plays a key role in the events contributing to obesity development and related dysfunction. In this work, we aimed at investigating whether changes in mRNA levels or DNA methylation of the A*NKRD26* gene occur in human obese individuals, and whether these changes correlate to altered levels of metabolic and inflammatory mediators in these subjects.

## Results

### Metabolic and inflammatory characterization of lean and obese subjects

Lean (*n* = 14) and obese (*n* = 20) adults of Caucasian ethnicity were recruited at the Federico II University of Naples and investigated in the present study. Each group included equal numbers of females and males. Clinical features of the two groups are shown in Tables 1 and 2. Serum TG (*p* < 0.001) and low-density lipoprotein cholesterol (LDL-C; *p* = 0.037) concentrations were increased and the serum levels of HDL-C (*p* = 0.002) were reduced in the obese compared with lean individuals. Also, the TG/HDL-C ratio, a described predictor of insulin resistance and cardiovascular risk [24–31], was increased in the obese subjects. No difference was found in either fasting blood glucose and serum total cholesterol (TC) levels between the two groups (Table 1). In addition, the obese individuals also featured an increase in serum level of C-reactive protein (CRP; *p* < 0.001; Table 1) as well as in several pro-inflammatory cytokines and chemokines (Table 2).

**Table 1:** Anthropometric and biochemical features of lean (*n* = 14; 7 males and 7 females) and obese (*n* = 20; 10 males and 10 females) individuals. For symmetrically distributed variables, data are shown as mean ± SD and statistical difference between the two groups was tested by two-tailed unpaired Student’s *t* test. For skewed distributions, data are shown as median (first quartile—Q1; third quartile—Q3) and statistical differences between the two groups were tested using Mann-Whitney *U* test. *BMI*, body mass index; *TC*, total cholesterol; *HDL-C*, high-density lipoprotein cholesterol; *LDL-C*, low-density lipoprotein cholesterol; *TG*, triglyceride; CRP, C-reactive protein

	Lean	Obese	*p* value
*n* (males/females)	14 (7/7)	20 (10/10)	
Age (years)	30.2 ± 2.6	37.0 ± 5.6	< 0.001
BMI (kg m^−2^)	22.3 ± 2.7	46.7 ± 6.9	< 0.001
Glucose (mg dL^−1^)	87.9 ± 7.1	95.4 ± 37.3	n.s.
TC (mg dL^−1^)	176.4 ± 33.4	179.1 ± 37.3	n.s.
HDL-C (mg dL^−1^)	63.9 ± 20.4	46.5 ± 10.2	0.002
LDL-C (mg dL^−1^)	94.9 ± 31.6	117.8 ± 29.2	0.037
TG (mg dL^−1^)	58.6 (43.4; 83.6)	119.5 (90.2; 162.0)	< 0.001
TG/HDL-C ratio	0.9 (0.6; 1.9)	2.6 (1.8; 4.2)	< 0.001
CRP (mg L^−1^)	0.2 (0.1; 0.4)	7.7 (1.7; 14.4)	< 0.001

**Table 2:** Pro-inflammatory cytokine and chemokine features of lean (*n* = 12; 6 males and 6 females) and obese (*n* = 18; 9 males and 9 females) individuals. Data are shown as median (first quartile—Q1; third quartile—Q3) and statistical differences between the two groups were tested using Mann-Whitney *U* test. *IL*, interleukin; *IFNγ*, interferon γ; *TNFα*, tumor necrosis factor α; *IP-10*, interferon gamma-induced protein 10; *MCP1*, monocyte chemotactic protein 1; *MIP1*, macrophage inflammatory protein 1; *RANTES*, regulated on activation, normal T cell expressed, and secreted

	Lean	Obese	*p* value
IL-1β (pg mL^−1^)	3.1 (2.7; 3.4)	3.4 (2.8; 4.5)	n.s.
IL-6 (pg mL^−1^)	4.1 (3.0; 5.3)	8.6 (6.0; 11.1)	< 0.001
IL-7 (pg mL^−1^)	9.0 (7.9; 12.0)	13.1 (8.8; 15.8)	n.s.
IL-9 (pg mL^−1^)	83.9 (59.0; 93.7)	83.9 (75.3; 96.7)	n.s.
IL-12 (pg mL^−1^)	25.6 (16.7; 40.1)	49.2 (34.9; 81.9)	0.006
IL-17 (pg mL^−1^)	125.9 (111.0; 149.4)	130.5 (116.9; 164.1)	n.s.
IFN-γ (pg mL^−1^)	101.2 (91.2; 113.9)	108.3 (85.4; 126.3)	n.s.
TNF-α (pg mL^−1^)	48.6 (39.7; 52.7)	47.2 (39.0; 54.7)	n.s.
IL-8 (pg mL^−1^)	16.5 (12.8; 18.2)	31.9 (22.0; 46.8)	< 0.001
Eotaxin (pg mL^−1^)	102.0 (63.8; 127.0)	116.3 (78.6; 157.6)	n.s.
IP-10 (pg mL^−1^)	313.3 (218.4; 850.3)	619.6 (528.5; 1095.1)	0.021
MCP-1 (pg mL^−1^)	7.0 (4.3; 14.5)	30.3 (18.3; 39.5)	0.001
MIP-1α (pg mL^−1^)	2.7 (2.6; 3.1)	3.2 (2.2; 4.5)	n.s.
MIP-1β (pg mL^−1^)	141.7 (90.5; 244.9)	202.6 (162.3; 424.4)	0.040
RANTES (pg mL^−1^)	21480.8 (15677.0; 24610.0)	28724.8 (26390.0; 36752.0)	< 0.001

### *ANKRD26* mRNA expression is reduced in peripheral blood leukocytes (PBL) from obese subjects

**Fig. 1:**
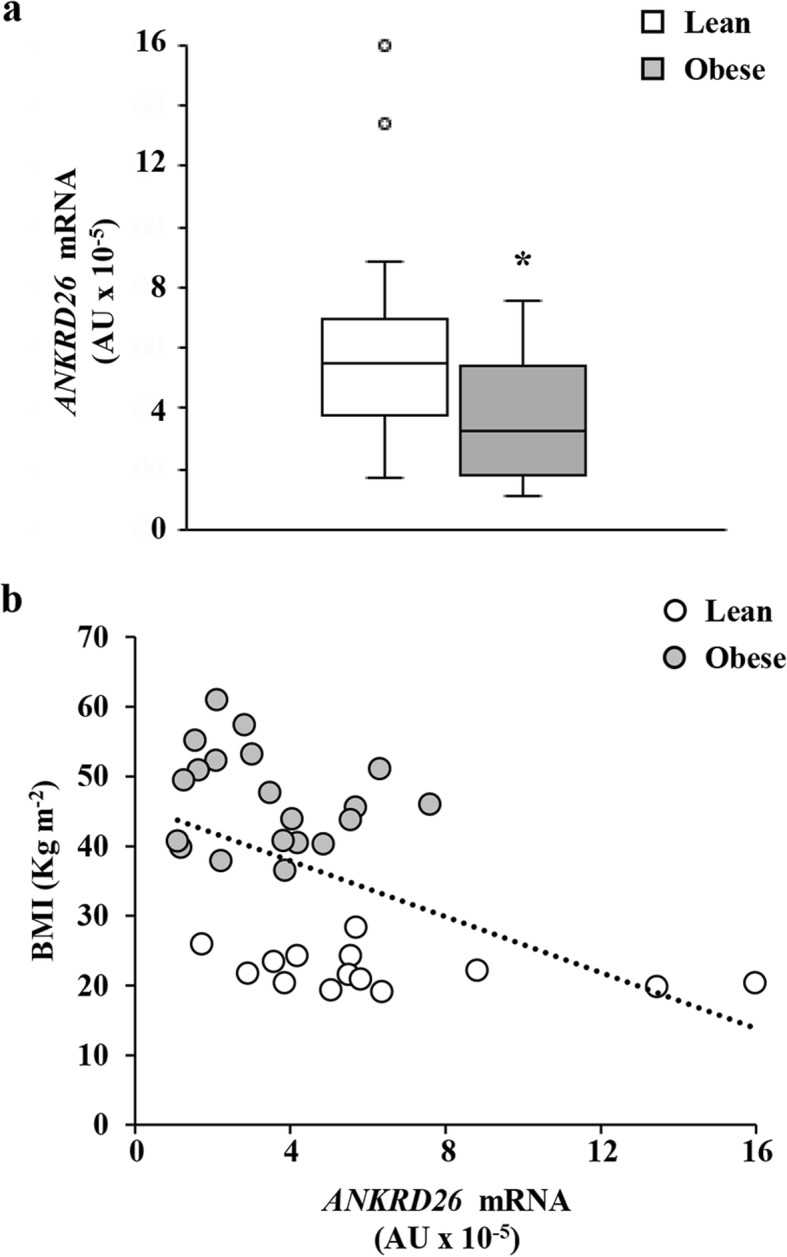
*ANKRD26* mRNA levels in PBL of lean and obese subjects. **a** *ANKRD26* mRNA levels were determined in lean (*n* = 14) and obese (*n* = 20) subjects. Values are expressed in absolute units (AU) and their distribution within each group is represented by box plots. Box plots show median (line within the box), quartiles (upper and lower box boundaries), and extreme values (whiskers). Statistical differences between the two groups were tested using nonparametric quantile regression, with inference based on median, to adjust for age. *p* = 0.027 vs. lean. **b** Relationship between BMI and *ANKRD26* gene expression was assessed by covariate-adjusted Spearman’s rank-order correlation adjusted for age. *n* = 34; *r* = − 0.494; *p* = 0.002

To determine whether changes of the *ANKRD26* gene expression occur in the PBL from the obese subjects, quantitative real-time PCR analysis was performed in cDNA from all of the subjects recruited in the study. We found a 40% reduction of the *ANKRD26* mRNA levels in PBL from the obese compared with the lean individuals (age-adjusted *p* = 0.027; Fig. 1a). Also, the *ANKRD26* gene transcriptional levels in PBL correlated positively to its mRNA expression in abdominal visceral adipose tissue (VAT; *n* = 10; *r* = 0.850; age-adjusted *p* = 0.014; Additional file 1: Figure S1). Within the obese group, the *ANKRD26* gene expression was much lower in the metabolically unhealthy (MUO) compared with the metabolically healthy obese (MHO) subjects, as defined in [32] (*p* = 0.004; Table 3). Also, *ANKRD26* mRNA levels in PBL correlated negatively to BMI (*n* = 34; *r* = − 0.494; age-adjusted *p* = 0.002; Fig. 1b), while no correlation was found between *ANKRD26* gene expression in the PBL and age (*n* = 34; *r* = − 0.226; *n.s.*).

**Table 3:** *ANKRD26* mRNA levels and methylation percentage of the CpG sites, − 689, − 659, and − 651, in MHO (*n* = 5) and MUO (*n* = 15) individuals. Obese subjects have been classified as MHO or MUO based on Wildman et al. [32]. In particular, metabolically unhealthy obese subjects are defined by the presence of two or more of these 6 criteria: blood pressure systolic ≥ 130 mmHg and/or diastolic ≥ 85 mmHg or use of anti-hypertensive drugs; fasting triglycerides ≥ 150 mg dl^−1^ or use of lipid-lowering drugs; fasting HDL-C ≤ 40 mg dL^−1^ in men and ≤ 50 mg dL^−1^ in women or use of lipid-lowering drugs; fasting glucose ≥ 100 mg dl^−1^ or use of anti-diabetic drugs; HOMA-IR > 90^th^ percentile; and CRP > 90^th^ percentile, while metabolically healthy obese subjects are defined by the presence of 0 or 1 of the previous criteria. For symmetrically distributed variables, data are shown as mean ± SD and the statistical difference between the two groups was tested by two-tailed unpaired Student’s *t* test. For skewed distributions, data are shown as median (first quartile—Q1; third quartile—Q3) and statistical differences between the two groups were tested using Mann-Whitney *U* test. *HDL-C*, high-density lipoprotein cholesterol; *TG*, triglyceride; *SBP*, systolic blood pressure; *DBP*, diastolic blood pressure; *CRP*, C-reactive protein; *HOMA-IR*, homeostatic model assessment of insulin resistance

	MHO	MUO	*p* value
Wildman criteria			
Glucose, mg dL^−1^	82.8 ± 8.3	99.6 ± 42.3	n.s.
HDL-C, mg dL^−1^	53.8 ± 13.6	44.4 ± 8.0	n.s.
SBP, mmHg	119.0 ± 10.2	135.3 ± 10.4	0.007
DBP, mmHg	84.0 ± 4.2	85.0 ± 6.0	n.s.
TG, mg dL^−1^	70.0 (62.2; 119.5)	137.0 (104.0; 170.0)	0.036
CRP, mg L^−1^	2.0 (0.5; 19.7)	9.1 (4.4; 17.2)	n.s.
HOMA-IR	1.6 (1.4; 2.2)	4.3 (3.0; 5.7)	0.006
*ANKRD26* mRNA, AU × 10^−5^	5.7 (4.4–7.0)	2.2 (1.6–3.8)	0.004
*CpG − 689*, % CpG methylation	36.7 ± 4.7	57.8 ± 15.7	0.009
*CpG − 569*, % CpG methylation	46.0 ± 8.9	51.2 ± 19.3	n.s.
*CpG − 651*, % CpG methylation	54.0 ± 16.7	62.6 ± 17.7	n.s.

### DNA methylation at the CpG sites, − 689, − 659, and − 651, is increased in PBL from obese subjects

**Fig. 2:**
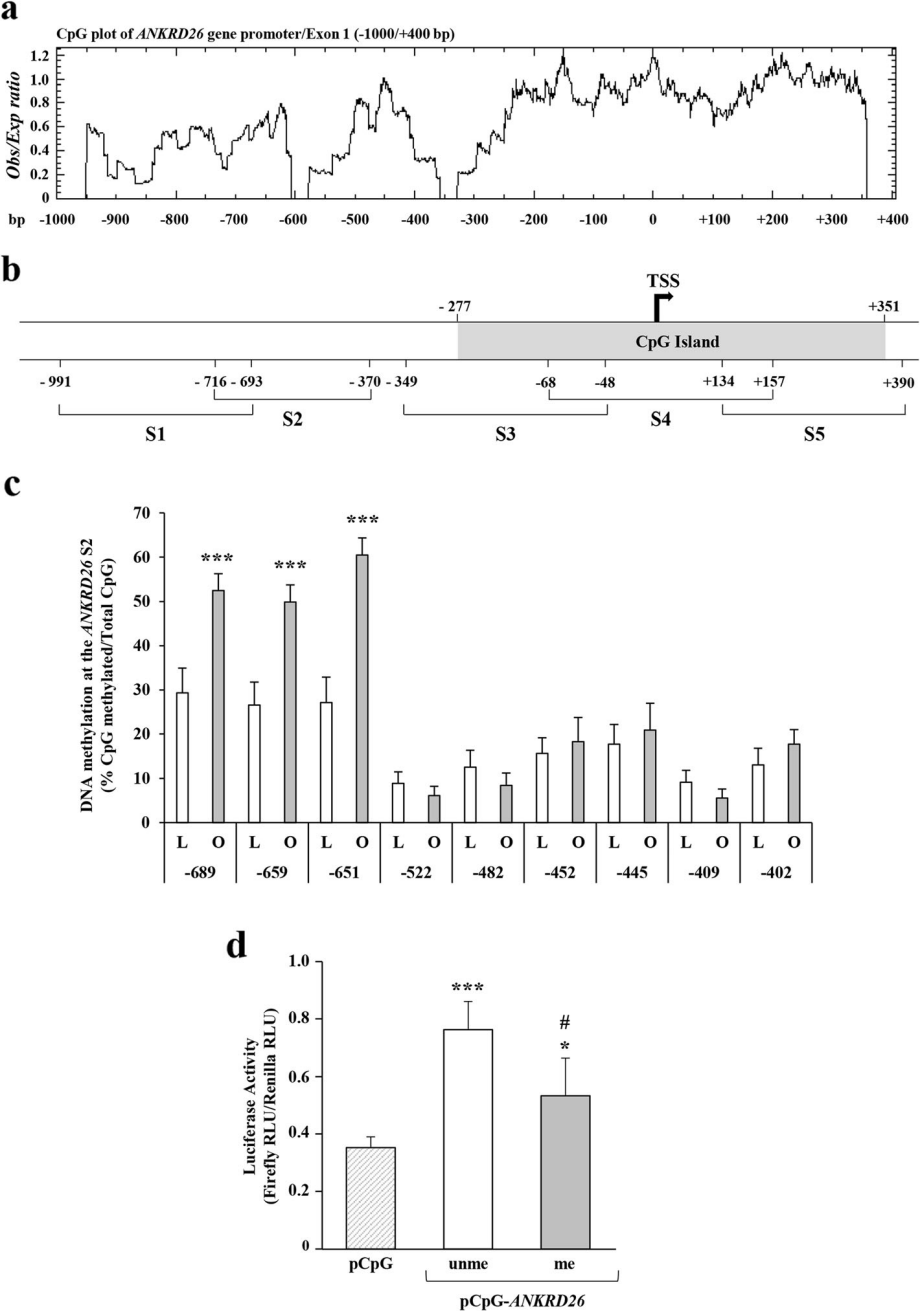
*ANKRD26* promoter methylation in PBL of lean and obese subjects and in vitro promoter activity. **a** A CpG plot of the *ANKRD26* gene promoter/Exon 1 (− 1000/+ 400 bp from TSS) was determined by using the EMBOSS CpGplot software. The presence of a 629 bp CpG island, straddling the *ANKRD26* TSS (− 277/+ 351 bp) and two regions enriched in CpGs, respectively, at − 900/− 600 bp and at − 580/− 370 bp upstream the TSS were identified. Values in *y*-axis are the minimum average observed (Obs) to expected (Exp) ratio of C plus G to CpG in a set of 10 windows that are required before a CpG island is reported. The minimum length, that a CpG island has to be before it is reported, is 200 bp. A window is set by default to 100 bp. Values in the *x*-axis are base pairs. **b** Schematic representation of the *ANKRD26* gene. Sub-region S1, − 991/− 693 bp; S2, − 716/− 370 bp; S3, − 349/− 48 bp; S4, − 68/+ 157 bp; S5, + 134/+ 390 bp. CpG Island, − 250/− 50. TSS, transcription start site; 5′-UTR, untranslated region. **c** Bisulfite sequencing methylation analysis of the *ANKRD26* S2 (CpG sites, − 689, − 659, − 651, − 522, − 482, − 452, − 445, − 409, − 402 bp from the TSS) in converted genomic DNA from PBL of 14 lean and 20 obese subjects. Results are means ± SD. Statistical difference between the means of the two groups was assessed by classical OLS regression model to adjust for age. ***p < 0.001 vs. lean % CpG methylation. **d** Luciferase activity of in vitro CpG methylated (me) or un-methylated (unme) pCpG-*ANKRD26* constructs and the pCpG empty vector was assessed as described in the “Methods” section. Firefly luciferase activity was normalized to Renilla luciferase activity. Luciferase activity is displayed in relative light units (RLU). Results are means ± SD from three independent experiments. Statistical difference between the means of the groups was assessed by one-way ANOVA. *p < 0.05 and ***p < 0.001 vs. pCpG empty vector; ^#^*p* < 0.05 vs. pCpG-*ANKRD26*unme

To investigate whether the downregulation of *ANKRD26* gene expression observed in the obese individuals is paralleled by changes in DNA methylation, we have initially performed a bioinformatics analysis of the *ANKRD26* promoter region. Based on EMBOSS CpGplot analysis, a 629 bp CpG island, straddling the *ANKRD26* transcription start site (TSS; − 277/+ 351 bp), was identified along with two regions enriched in CpG sites, respectively, at − 900/− 600 bp and at − 580/− 370 bp upstream the TSS (Fig. 2a). An *ANKRD26* gene promoter/Exon 1 region (− 991/+ 390 bp), embedding the CpG island and the CpG enriched regions, was thus divided in 5 consecutive sub-regions, which were termed S1 (− 991/− 693 bp), S2 (− 716/− 370 bp), S3 (− 349/− 48 bp), S4 (− 68/+ 157 bp), and S5 (+ 134/+ 390 bp) (Fig. 2b), and a direct analysis of cytosine methylation at this region was subsequently performed by sequencing of bisulfite-converted DNA obtained from PBL of 3 individuals featuring the lowest and 3 featuring the highest BMI. In lean subjects, the bisulfite sequencing analysis of the *ANKRD26* gene promoter/Exon 1 region revealed a high-density DNA hyper-methylation at the sub-region 1 (S1; ~ 85%), a low/moderate CpG methylation at the S2 (~ 20%), and a massive DNA hypo-methylation at the sub-regions S3, S4, and S5 (Additional file 1: Table S1). Similar CpG methylation patterns were also observed at the sub-regions S1, S3, S4, and S5 of the *ANKRD26* gene promoter/Exon 1 region in the obese individuals, while, in these patients, a significantly increased DNA methylation at the S2 (~ 27%; *p* < 0.05) was found (Additional file 1: Table S1).

Based on these results, the DNA methylation pattern at the S2 was further investigated by extending sequencing analysis to bisulfite-converted DNA obtained from the PBL of 11 further lean and 17 further obese subjects. This additional sequencing revealed a 40–80% increased density of the DNA methylation at 3 close CpG dinucleotides (− 689, − 659, and − 651 bp from the *ANKRD26* TSS) in the obese compared with lean subjects (Fig. 2c). DNA methylation at the − 689, − 659, and − 651 bp CpG sites was increased by about 1.8-fold (age-adjusted *p* < 0.001), 1.9-fold (age-adjusted *p* = 0.040), and 2.2-fold (age-adjusted *p* = 0.025), respectively, in the obese compared with the lean (Fig. 2c). No relevant differences of DNA methylation were observed among normal weight and obese subjects at the CpG sites, − 522, − 482, − 452, − 445, − 409, and − 402 bp (Fig. 2c). Also, within the obese group, the CpG site − 689 bp was hyper-methylated in the MUO compared with the MHO subjects (*p* = 0.009; Table 3), while no differences in CpG methylation were observed at the sites 659 and − 651 bp (Table 3).

### Methylation at the CpGs, − 689, − 659, and − 651, modulates *ANKRD26* promoter activity in vitro

**Fig. 3:**
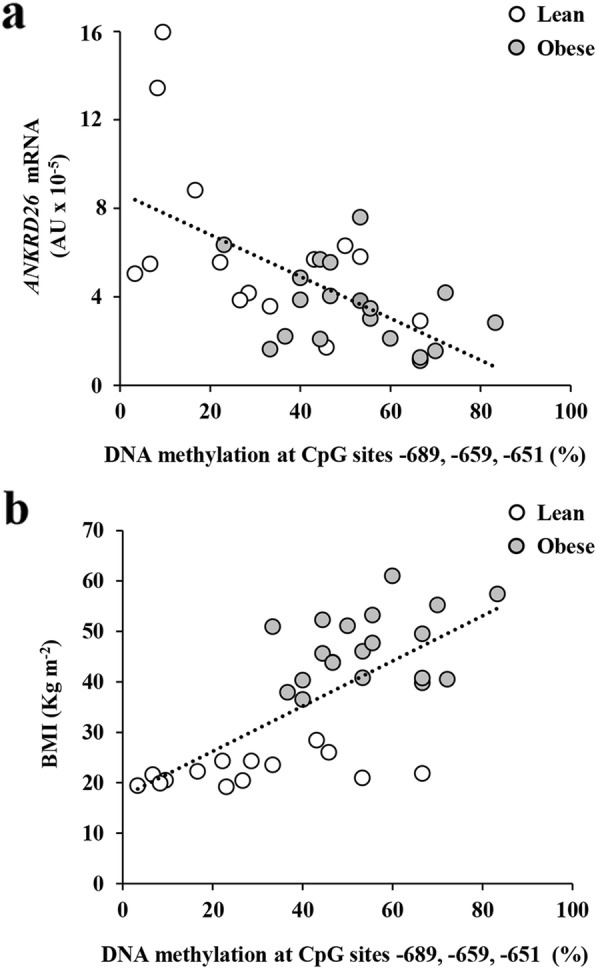
*ANKRD26* gene expression, promoter methylation, and BMI. **a** Relationship between *ANKRD26* gene expression and the combined DNA methylation at the CpGs − 689, − 659, and − 651 was assessed in lean (*n* = 14) and obese individuals (*n* = 20) by covariate-adjusted Spearman’s rank-order correlation adjusted for age. *r* = − 0.539, age-adjusted *p* < 0.001. **b** Relationship between BMI and the combined DNA methylation at the CpGs − 689, − 659, and − 651 was assessed in lean (*n* = 14) and obese individuals (*n* = 20) by covariate-adjusted Spearman’s rank-order correlation adjusted for age. *r* = 0.736, age-adjusted *p* < 0.001

To identify a potential cause-effect relationship between the DNA methylation at the *ANKRD26* gene promoter and its gene transcription, a DNA fragment (− 716/− 597 bp) containing the CpG sites, − 689, − 659, and − 651 bp, was cloned in a pCpG-free vector. Luciferase activities of either the in vitro CpG methylated or the un-methylated pCpG-*ANKRD26* luciferase reporter vectors were then assayed in human embryonic kidney HEK293 cells. As shown in Fig. 2d, the un-methylated *ANKRD26* promoter region induced a 2.2-fold increase of luciferase activity compared with the empty vector, indicating that this fragment features promoter activity. On the other hand, the methylation of the three selected CpG sites at the *ANKRD26* promoter causes a 30% reduction of the luciferase activity compared with the un-methylated pCpG-*ANKRD26* luciferase vector, indicating that methylation of one or more of these 3 CpG dinucleotides in vitro represses the *ANKRD26* gene transcription. Also, a negative correlation was found between the combined methylation percentage at the cytosine residues − 689, − 659, and − 651 of the *ANKRD26* promoter and the expression levels of the *ANKRD26* gene in PBL (*n* = 34, *r* = − 0.539, age-adjusted *p* < 0.001; Fig. 3a). The combined methylation of these 3 CpG sites positively correlated to BMI (*n* = 34, *r* = 0.736, age-adjusted *p < 0.001*; Fig. 3b). No correlation was found between the combined DNA methylation at these 3 CpG sites and age (*n* = 34, *r* = − 0.223, *n.s*). It is noteworthy that similar results were obtained when these relationships with *ANKRD26* mRNA expression, BMI, and age were performed on individual CpG methylation percentages (Additional file 1: Table S2).

### Both *ANKRD26* mRNA expression and CpG methylation associate with obesity-related endophenotypes

We then explored whether changes of the *ANKRD26* gene expression correlate to metabolic and inflammatory marks. As reported in Table 4, we found that *ANKRD26* mRNA levels correlated inversely to serum TG concentration (*n* = 34, *r* = − 0.630, age-adjusted *p* < 0.001) and directly to serum HDL-C concentration (*n* = 34, *r* = 0.647, age-adjusted *p* < 0.001). In addition, the *ANKRD26* gene expression correlated negatively to serum levels of CRP (*n* = 34, *r* = − 0.494, age-adjusted *p* < 0.001), interleukin 1β (IL-1β; *n* = 30, *r* = − 0.498, age-adjusted *p* = 0.010), IL-6 (*n* = 30, *r* = − 0.401, age-adjusted *p* = 0.027), IL-12 (*n* = 30, *r* = − 0.506, age-adjusted *p* = 0.002), IL-8 (*n* = 30, *r* = − 0.650, age-adjusted *p* < 0.001), interferon gamma-induced protein 10 (IP-10; *n* = 30, *r* = − 0.520, age-adjusted *p* = 0.002), macrophage inflammatory protein 1α (MIP-1α; *n* = 30, *r* = − 0.513, age-adjusted *p* = 0.006), MIP-1β (*n* = 30, *r* = − 0.601, age-adjusted *p* < 0.001), and normal T cell expressed and secreted (RANTES; *n* = 30, *r* = − 0.476, age-adjusted *p* = 0.002), as well. Interestingly, as also reported in Table 5, the combined DNA methylation levels of the CpG dinucleotides − 689, − 659, and − 651 bp at the *ANKRD26* promoter correlated positively to serum TG concentration (*n* = 34, *r* = 0.593, age-adjusted *p* < 0.001) and negatively to serum HDL-C concentration (*n* = 34, *r* = − 0.539, age-adjusted *p* < 0.001). Also, the combined methylation of these 3 CpG sites correlate directly to serum levels of CRP (*n =* 30, *r* = 0.524, age-adjusted *p* = 0.001), IL-6 (*n* = 30, *r* = 0.577, age-adjusted *p* = 0.002), IL-12 (*n* = 30, *r* = 0.453, age-adjusted *p* = 0.013), IL-8 (*n* = 30, *r* = 0.698, age-adjusted *p* = 0.001), and RANTES (*n* = 30, *r* = 0.514, age-adjusted *p* = 0.013). Very interestingly, these relationships were also confirmed when these biochemical and pro-inflammatory parameters were associated to individual CpG methylation percentages (Additional file 1: Table S2).

**Table 4:** *ANKRD26* gene expression in relation to metabolic and inflammatory parameters. Relationship between metabolic or inflammatory parameters and *ANKRD26* gene expression was assessed in lean and obese individuals by covariate-adjusted Spearman’s rank-order correlation adjusted for age. Number of individuals (*n*), correlation coefficient *r*, and age-adjusted *p* value are shown in the table. *TG*, triglyceride; *TC*, total cholesterol; *HDL-C*, high-density lipoprotein cholesterol; *LDL-C*, low-density lipoprotein cholesterol; *CRP*, C-reactive protein; *IL*, interleukin; *IFNγ*, interferon γ; *TNFα*, tumor necrosis factor α; *IP-10*, interferon gamma-induced protein 10; *MCP1*, monocyte chemotactic protein 1; *MIP1*, macrophage inflammatory protein 1; *RANTES*, regulated on activation, normal T cell expressed, and secreted

Parameters	Gene expression		
	*n*	*r*	*p* value
Glucose	34	− 0.233	n.s.
TG	34	− 0.630	< 0.001
TC	34	− 0.113	n.s.
HDL-C	34	0.647	< 0.001
LDL-C	34	− 0.315	n.s.
CRP	34	− 0.494	0.001
IL-1β	30	− 0.498	0.010
IL-6	30	− 0.401	0.027
IL-7	30	− 0.185	n.s.
IL-9	30	− 0.123	n.s.
IL-12	30	− 0.506	0.002
IL-17	30	− 0.085	n.s.
IFN-γ	30	− 0.112	n.s.
TNF-α	30	− 0.037	n.s.
IL-8	30	− 0.650	< 0.001
Eotaxin	30	− 0.116	n.s.
IP-10	30	− 0.520	0.002
MCP-1	30	− 0.206	n.s.
MIP-1α	30	− 0.513	0.006
MIP-1β	30	− 0.601	< 0.001
RANTES	30	− 0.476	0.002

**Table 5:** Combined DNA methylation at the CpG sites, − 689, − 659, and − 651 of the *ANKRD26* promoter in relation to metabolic and inflammatory parameters. Relationships were assessed in lean and obese individuals by covariate-adjusted Spearman’s rank-order correlation adjusted for age. Number of individuals (*n*), correlation coefficient *r*, and age-adjusted *p* value are shown on the table. *TG*, triglyceride; *TC*, total cholesterol; *HDL-C*, high-density lipoprotein cholesterol; *LDL-C*, low-density lipoprotein cholesterol; *CRP*, C-reactive protein; *IL*, interleukin; *IFNγ*, interferon γ; *TNFα*, tumor necrosis factor α; *IP-10*, interferon gamma-induced protein 10; *MCP1*, monocyte chemotactic protein 1; *MIP1*, macrophage inflammatory protein 1; *RANTES*, regulated on activation, normal T cell expressed, and secreted

Parameters	Combined % CpG methylation		
	*n*	*r*	*p* value
Glucose	34	0.081	n.s.
TG	34	0.593	< 0.001
TC	34	0.155	n.s.
HDL-C	34	− 0.539	0.004
LDL-C	34	0.376	0.044
CRP	34	0.524	0.001
IL-1β	30	0.242	n.s.
IL-6	30	0.577	0.002
IL-7	30	0.182	n.s.
IL-9	30	0.032	n.s.
IL-12	30	0.453	0.013
IL-17	30	− 0.001	n.s.
IFN-γ	30	− 0.025	n.s.
TNF-α	30	− 0.083	n.s.
IL-8	30	0.698	0.001
Eotaxin	30	0.001	n.s.
IP-10	30	0.337	n.s.
MCP-1	30	0.305	n.s.
MIP-1α	30	0.333	0.050
MIP-1β	30	0.377	n.s.
RANTES	30	0.514	0.013

### Both *ANKRD26* mRNA expression and CpG methylation associate with TG/HDL-C ratio

**Fig. 4:**
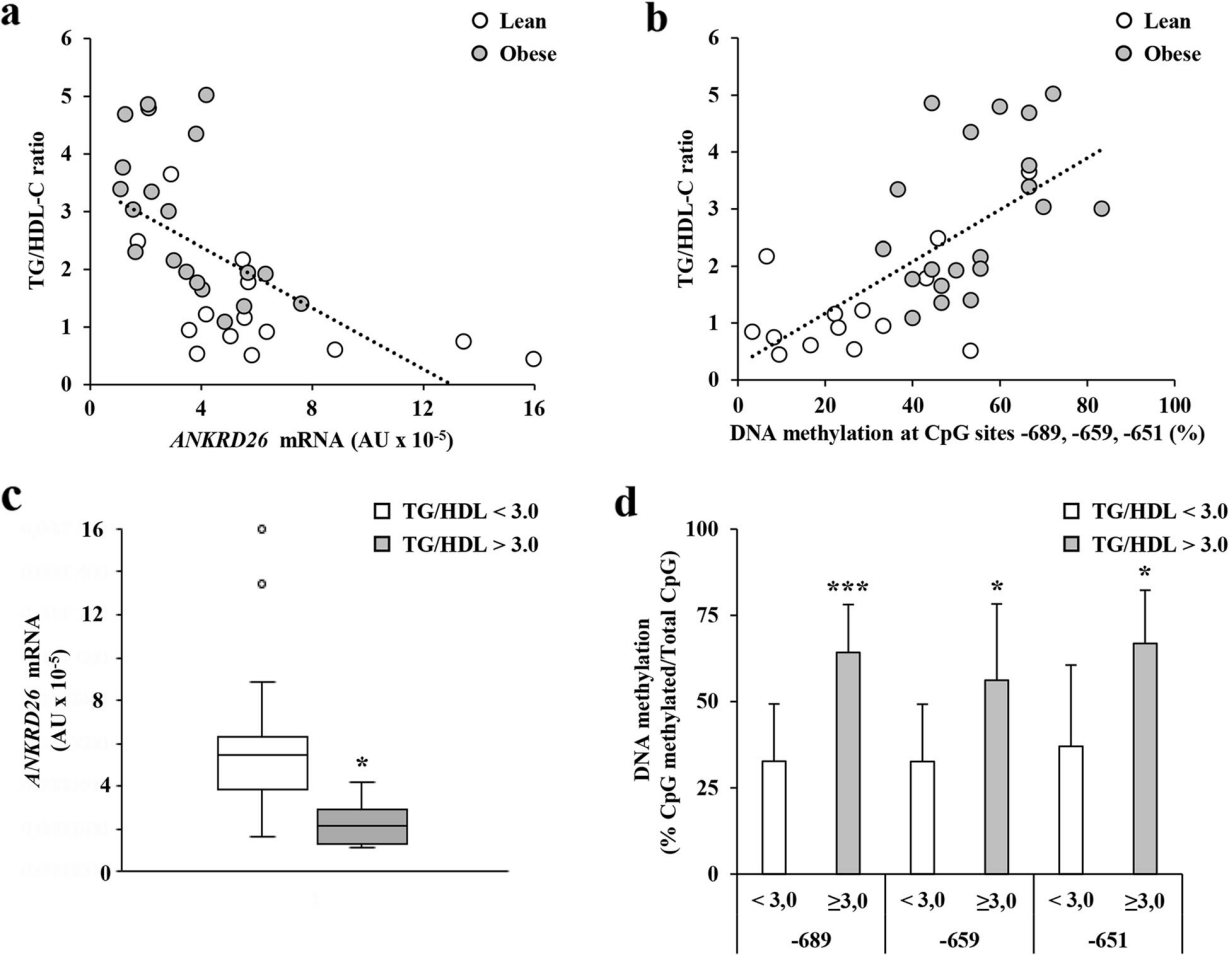
*ANKRD26* gene expression, promoter methylation, and cardio-metabolic risk. **a** Relationship between *ANKRD26* expression and TG/HDL-C ratio was assessed in lean (*n* = 14) and obese individuals (*n* = 20) by covariate-adjusted Spearman’s rank-order correlation adjusted for age (*n* = 34; *r* = − 0.728; *p* < 0.001). **b** Relationships between TG/HDL-C ratio and the combined DNA methylation at the CpGs − 689, − 659, and − 651 in lean (*n* = 14) and obese individuals (*n* = 20) was assessed by covariate-adjusted Spearman’s rank-order correlation adjusted for age. *r* = 0.666, *p* < 0.001. **c** *ANKRD26* mRNA levels in 11 individuals with the TG/HDL-C > 3.0 and in 23 individuals with the TG/HDL-C < 3.0. Values are expressed in absolute units (AU) and their distribution within each group is represented by box plots. Box plots show median (line within the box), quartiles (upper and lower box boundaries), and extreme values (whiskers). Statistical differences between the two groups were tested using nonparametric quantile regression, with inference based on median, to adjust for BMI. *p* = 0.016 vs. individuals with the TG/HDL-C < 3.0. **d** Bisulfite sequencing methylation analysis of the *ANKRD26* CpG sites, − 689, − 659, and − 651 bp from the TSS in converted genomic DNA from PBL of 11 individuals with TG/HDL-C > 3.0 and in 23 individuals with the TG/HDL-C < 3.0. Results are means ± SD. Statistical difference between the means of the two groups was assessed by classical OLS regression model to adjust for BMI. *p < 0.05 and ***p < 0.001 vs. individuals with TG/HDL-C < 3.0

Interestingly, a negative correlation between the *ANKRD26* gene expression and TG/HDL-C ratio was also noted (*n* = 34, *r* = − 0.728, age-adjusted *p* < 0.001; Fig. 4a). Also, the percentage of CpG dinucleotides methylation at − 689, − 659, and − 651 bp, taken as combined or as individual CpG methylation, of the *ANKRD26* promoter positively correlated to the TG/HDL-C ratio (*n* = 34, *r* = 0.666, age-adjusted *p* < 0.001; Fig. 4b and Additional file 1: Table S2). When the study group subjects were stratified based on the TG/HDL-C ratio [24], we found that, independent of BMI, the *ANKRD26* mRNA levels were reduced by about 60% in the individuals with the TG/HDL-C ratio > 3.0 compared with subjects with a TG/HDL-C ratio < 3.0 (BMI-adjusted *p* = 0.016; Fig. 4c). Also, DNA methylation at the − 689, − 659, and − 651 bp CpG sites was increased by about 2.0-fold (BMI-adjusted *p* < 0.001), 1.7-fold (BMI-adjusted *p* = 0.040), and 1.8-fold (BMI-adjusted *p* = 0.025), respectively, in the individuals with a TG/HDL-C ratio > 3.0 compared with subjects featuring a TG/HDL-C ratio below the cutoff value (Fig. 4d).

## Discussion

The epigenome, at the interface between genome and the environment, undergoes continuous changes through the individual lifetime, which may have major implications to disease susceptibility [7–9]. Consistently, mounting evidence indicates an important role of the epigenetic modifications in determining risk of obesity [4, 33, 34]. Thus, the identification of epigenetic profiles associated with obesity may provide important novel insight into disease pathogenesis, biomarker discovery, and pharmacological targets.

In this report, we have demonstrated that the *ANKRD26* gene expression is downregulated in human PBL from obese compared with lean subjects. We also found that the *ANKRD26* gene expression in the blood cells correlates positively to its mRNA expression in abdominal VAT, a tissue relevant to ANKRD26 protein function [14, 22, 35, 36], suggesting that perturbation of *ANKRD26* expression is biologically relevant to obesity. We also report that (i) hyper-methylation at the CpG dinucleotides − 689, − 659, and − 651 bp from the *ANKRD26* TSS occurs in PBL from the obese compared with the lean controls; (ii) a direct cause-effect relationship exists between these methylation events and *ANKRD26* gene expression; and (iii) BMI correlates to both gene expression and DNA methylation of the *ANKRD26* gene in PBL. Altogether, these findings indicate that hyper-methylation at the *ANKRD26* promoter and the accompanying downregulation of gene expression are common features of obesity in humans. DNA methylation is the most common and better-characterized epigenetic modification [37, 38]. Evidence in literature also supports the hypothesis that it might also be transgenerationally inherited [39]; however, DNA methylation often occurs in response to environmental variability [40]. Therefore, whether the observed epigenetic downregulation of the *ANKRD26* gene precedes or is a consequence of obesity and thus, whether the increased CpG methylation at the *ANKRD26* promoter has a role in the regulation of BMI and/or is predictive of obesity onset deserves to be further investigated. Changes in DNA methylation at this gene in humans are consistent with our previous findings in murine models of diet-induced obesity. Indeed, persistence of the high-fat diet regimen and the onset of obesity in mice causes epigenetic silencing of the *Ankrd26* mRNA expression due to the specific hyper-methylation of the *Ankrd26* promoter at the CpG sites − 436 and − 431 bp [17]. This supports the hypothesis that similar changes of DNA methylation, occurring in the human *ANKRD26* gene, might be the result of increased BMI/adiposity typical of obesity. Furthermore, our finding reveal a more pronounced silencing of *ANKRD26* and increased DNA methylation at the − 689 bp CpG in the MUO compared with the MHO individuals. This supports the hypothesis that the epigenetic regulation of the *ANKRD26* gene may be sensitive to the alteration of the metabolic and inflammatory profile occurring in the metabolically unhealthy obese [32].

We have further explored the potential clinical relevance of the epigenetic silencing of *ANKRD26* by correlating both the *ANKRD26* gene expression and DNA methylation to obesity-related endophenotypes. In particular, we have reported that both *ANKRD26* mRNA and DNA methylation levels of the CpG dinucleotides − 689, − 659, and − 651 bp, taken as combined or as single CpG methylation percentage, correlate to serum TG and HDL-C concentration. In line with our previous findings in abdominal VAT from obese subjects with normal glucose tolerance [17], we also found that changes of *ANKRD26* gene expression and methylation correlate to increased levels of the inflammatory cytokine IL-6, the chemokines IL-8, and RANTES [41], and with CRP, a sensitive marker of low-grade inflammation and cardio-metabolic risk in different populations [42–46]. In addition, perturbations of *ANKRD26* gene expression and methylation correlate to the TG/HDL-C ratio, a recently identified mark of cardio-metabolic risk and of insulin resistance [24–31]. Importantly, stratification of the study group based on the TG/HDL-C index revealed association of higher levels of *ANKRD26* methylation and reduced gene expression with high-cardio-metabolic risk, independent of BMI, indicating that alterations of both the DNA methylation and mRNA expression at the *ANKRD26* gene correlate to increased risk of insulin resistance and cardiovascular disease in humans.

The major strength of our research design was the use of a site-by-site approach for the investigation of DNA methylation within the *ANKRD26* gene promoter/Exon 1 (− 991/+ 390 bp from TSS). This allowed us to (i) screen the cytosine methylation of 92 CpG dinucleotides related to our candidate gene, whose detection, at the best of our knowledge, is only partially covered by commercial DNA methylation arrays and (ii) identify among lean and obese subjects’ specific methylation changes at the CpGs, − 689, − 659, and − 651, which are relevant for the *ANKRD26* gene expression regulation and are associated with pro-inflammatory and cardio-metabolic risk factors, and which cannot be otherwise discovered. However, some limitations exist in the current study starting from the limited sample size of the recruited individuals. Our findings should be therefore replicated in other large and independent population. Nevertheless, in support of our results, *ANKRD26* has been included in a dataset of loci, identified through a whole transcript-based array, where its gene expression negatively correlated with BMI in a study conducted on the subcutaneous WAT from a male Swedish and Danish discovery cohort (*n* = 96) [47].

## Conclusion

In conclusion, this study demonstrates that downregulation of the *ANKRD26* gene caused by promoter hyper-methylation at specific CpGs represents a common abnormality in obese patients. These changes correlate to the pro-inflammatory profile and the cardio-metabolic risk factors of the obese individuals, suggesting they mark the adverse health outcome occurring in some of these patients.

## Methods

### Subject enrollment, sampling, and management

#### Subject enrollment

Thirty-four individuals, 14 lean (7 males and 7 females) undergoing cholecystectomy and 20 obese (10 males and 10 females) undergoing sleeve gastrectomy, were recruited at the Federico II University of Naples, and defined, respectively, based on BMI < 25 kg m^−2^ and BMI ≥ 30 kg m^−2^ [48]. Participants were selected as follows. Inclusion criteria are as follows: age between 25 and 50 years and Caucasian ethnicity. Exclusion criteria are as follows: not knowledge of a verifiable medical treatment period; psychotic disorders; severe depression; personality and eating behavior disorders assessed by a dedicated psychiatrist or psychologist; alcoholism and drug addiction; diseases related to reduced life expectancy; inability to take care of him/herself; inadequate family and social support; obesity secondary to endocrinopathies; gastrointestinal inflammatory diseases; *risk* of *upper gastrointestinal tract bleeding*; previous or current tumors; and use of drugs that can influence epigenetic status. The study adhered to the Code of Ethics of the World Medical Association (Declaration of Helsinki) and has been reviewed and approved by the Ethic Committee of the Federico II University of Naples (Ethics Approval Number: No. 254/17). Informed consent was obtained individually from all of the subjects enrolled in the study. Obese subjects have been also classified as MHO or MUO based on Wildman et al. [32]. In particular, MUO is defined in subjects by the presence of two or more of these 6 criteria: blood pressure systolic ≥ 130 mmHg and/or diastolic ≥ 85 mmHg or use of anti-hypertensive drugs; fasting triglycerides ≥ 150 mg dL^−1^ or use of lipid-lowering drugs; fasting HDL-C ≤ 40 mg dL^−1^ in men and ≤ 50 mg dL^−1^ in women or use of lipid-lowering drugs; fasting glucose ≥ 100 mg dL^−1^ or use of anti-diabetic drugs; homeostatic model assessment of insulin resistance (HOMA-IR) > 90^th^ percentile; and CRP > 90^th^ percentile, while MHO subjects are defined by the presence of 0 or 1 of the previous criteria.

#### Sampling and management

Blood and sera samples were collected from fasted patients the day before surgery, and successively handled, processed, and stored by the same investigator. In detail, 1 whole blood vacuum tube was collected from each subject (lean, *n* = 14; obese, *n* = 20) enrolled in the study for PBL isolation. Also, 1 serum vacuum tube was collected from each subject (lean, *n* = 14; obese, *n* = 20) enrolled in the study for the determination of biochemical parameters, while a further serum vacuum tube was collected from 30 patients out of 34 for the determination of inflammatory parameters through Bioplex multiplex analysis. Blood cell count was evaluated for each individual patient and no significant differences were reported within subjects. PBL isolation was performed on each sample by using the following procedure. Whole blood samples were incubated on ice for 15 min with 5 volume of erythrocyte lysis buffer (KHCO_3_ 10 mM, NH_4_Cl 155 mM, EDTA 0.1 mM), and PBL was recovered by centrifugation at 400×*g* for 10 min, and then stored at − 80 °C in RNA Later stabilization reagent (Qiagen, Hilden, Germany). At the end of the recruitment, PBL obtained from each patient were lysed in RLT buffer 1× and total RNA and genomic DNA were then isolated using the AllPrep DNA/RNA/miRNA Universal kit (Qiagen) and following the manufacturer’s instructions.

#### Abdominal VAT sampling

Abdominal VAT biopsies were collected from 10 of the recruited patients (5 lean and 5 obese) and were handled and stored by the same investigator. In detail, biopsies were snap frozen in liquid nitrogen, and then stored at − 80 °C in RNA Later stabilization reagent (Qiagen). At the end of the recruitment, VAT biopsies were homogenized in RLT buffer 1× by using the Tissue Lyser LT bead mill (Qiagen), and total RNA was then isolated using the AllPrep DNA/RNA/miRNA Universal kit (Qiagen) and following the manufacturer’s instructions.

### Determination of biochemical and inflammatory parameters

Plasma glucose and serum levels of TC, HDL-C, LDL-C, TG, and CRP were determined with an ABX Pentra 400 (Horiba Ltd., Kyoto, Japan). Serum samples have been also used for the dosage of the following secreted inflammatory mediators by Bioplex multiplex Human Cytokine, Chemokine and Growth factor kit (Bio-Rad Laboratories, Hercules, CA): IL-1β, IL-6, IL-7, IL-9, IL-12, IL-17, Interferon γ (IFNγ), tumor necrosis factor α (TNFα), IL-8, eotaxin, IP-10, MCP1, MIP1α, MIP1β, and RANTES.

### Quantitative real-time PCR

cDNA synthesis was performed from total RNA (1 μg) isolated from PBL samples or from abdominal VAT biopsies by using the SuperScript™ III Reverse Transcriptase (Thermo Fisher Scientific, Waltham, MA) and following the manufacturer’s instructions. cDNA from each sample was then used as a template for quantitative real-time PCR assays. In detail, for each sample, reactions were performed in triplicates using iQ SYBR Green Supermix on an iCycler real-time detection system (Bio-Rad Laboratories) by using the following cycling condition and reaction protocol. *Cycling condition*: 10 min at 95 °C for 1 cycle, and 15 s at 95 °C and 1 min at 60 °C repeated for 40 cycles. *Reaction protocol*: cDNA (25 ng), forward and reverse PCR primers (200 nM each), iQ SYBR Green Supermix 1X (Bio-rad laboratories) in a final volume of 10 μL. The absolute quantification of the hs*ANKRD26* mRNA expression levels for each sample was carried out by using hs*28S* ribosomal RNA, as control reference [49]. hs*28S* ribosomal RNA has been selected as reference gene upon a comparison with other three reference genes of the threshold cycle (Ct) variability (Additional file 1: Table S3). The following standard curves were used for the calculations: hs*ANKRD26* standard curve, *y* = − 3.661 × + 36.554, *r* = 0.9995, and hs*28S* standard curve, *y* = − 3.587 × + 36.164, *r* = 0.9990. Primer sequences were as follow: hs*28S* F: 5′-cccagtgctctgaatgtcaa-3′; hs*28S* R: 5′-agtgggaatctcgttcatcc-3′; hs*ANKRD26* F: 5′-gtatgctagtagtggtcctgc-3′; and hs*ANKRD26* R: 5′-gtaggccttccttcatcctcat-3′.

### DNA methylation analysis by bisulfite conversion

Genomic DNA from PBL and from abdominal VAT biopsies was prepared as described above. Bisulfite treatment of 350 ng of genomic DNA for each sample was converted with the EZ DNA Methylation Kit (Zymo Research, Orange, CA), following the manufacturer’s instructions. For the analysis, *ANKRD26* promoter was divided as follows: S1, − 786/− 722 bp; S2, − 716/− 370 bp; S3, − 349/− 48 bp; S4, − 68/+ 147 bp; and S5, + 134/+ 390 bp from TSS. Bisulfite-converted genomic DNA was amplified by PCR using specific primers for each site. The PCR fragments were then cloned into the pGEM T-Easy vector system (Promega, Madison, WI). Bisulfite genomic sequencing was performed as previously reported [50]. In detail, to determine methylation status, 10 clones for each sample were sequenced on AB 3500 genetic analyzer (Life Technologies, Carlsbad, CA). The percentage of individual methylation and the percentage of combined methylation at the CpGs − 689, − 659, and − 651 were calculated using the following two formulas: individual methylation % = (CpG methylated. CpG total^−1^) × 100; combined methylation % = (% methylation of CpG − 689 + % methylation of CpG − 659 + % methylation of CpG − 651)/3. Primers sequences: *ANKRD26* S1 F: 5′-gtaatttttgttgagattttatttga-3′, *ANKRD26* S1 R: 5′-actacaatctccacctcctaaactc-3′, *ANKRD26* S2 F: 5′-agtttaggaggtggagattgtagtg-3′, *ANKRD26* S2 F: 5′-acaaatacaacaacaaaaaacacaaa-3′, *ANKRD26* S3 F: 5′-gtatttaaagggatatggaaggg-3′, *ANKRD26* S3 R: 5′-cccaataatcaaatatactccatac-3′, *ANKRD26* S4 F: 5′-tggagtatatttgattattgggtttt-3′, *ANKRD26* S4 R: 5′-aacttcaaaaacacctcatatctctct-3′, *ANKRD26* S5 F: 5′-agagagatatgaggtgtttttgaagtt-3′, *ANKRD26* S5 R: 5′-caaaccattcttcctaaacaaaaaa-3′. Bioinformatics analysis was carried out using EMBOSS CpGplot (available from www.ebi.ac.uk/Tools/seqstats/emboss_cpgplot/; accessed November 2015).

### Cloning, in vitro methylation, and luciferase assay

*ANKRD26* promoter (− 716/− 597 bp) was amplified by PCR. The purified PCR fragment was cloned into the firefly luciferase reporter pCpG-free-promoter-Lucia vector (Invivogen, Toulouse, France). This vector is completely void of CpG dinucleotides in all the elements required for replication and selection of the plasmid in *E. coli* and gene expression in mammalian cells. Also, it is devoid of all Dam methylation sites (GATC) to prevent prokaryotic methylation [51]. The pCpG-*Ankrd26* vector was amplified in *E. coli* GT115 cells (Invivogen). In vitro methylation was performed using the M.SssI CpG methyltransferase following manufacturer’s protocol (New England BioLabs, Ipswich, MA). Un-methylated DNA was obtained in the absence of M.SssI. Methylation was confirmed by digestion with MspJI (New England BioLabs). HEK-293 cells were transfected with the CpG methylated or un-methylated pCpG-*ANKRD26* vector and Renilla control vector (Promega, Madison WI) by lipofectamine (Life Technologies), following manufacturer’s instructions. Firefly luciferase activity of each transfection was normalized for transfection efficiency against Renilla luciferase activity.

### Sample size

Sample sizes were calculated by using the G*Power 3.1.9.2 software (Heinrich-Heine-Universität Düsseldorf, Germany) [52]. In detail, a sample size of 34 participants, *n* = 20 for obese (group 1) and *n* = 14 for lean (group 2), achieved 95% power to detect a difference of – 39 × 10^−5^ between the null hypothesis that both group means are equal to 159 × 10^−5^ and the alternative hypothesis that the mean of group 2 is different and equal to 198 × 10^−5^, considering a two-sample *t* test, a significance level (α) of 0.05, an estimated group standard deviations of 38 × 10^−5^ and 18 × 10^−5^, and an allocation ratio N2/N1 = 1.4. For calculations, values of the *Ankrd26* mRNA (mean ± SD) in obese and lean mice were applied [17]. Information about *ANKRD26* mRNA levels in humans, to the best of our knowledge, was not available. A 1.4 allocation ratio N2/N1 was decided based on the prediction of a 40% larger recruitment of obese versus lean individuals.

### Statistical and experimental procedures

#### Statistical procedures

For symmetrically distributed variables, data are shown as mean ± SD, while for skewed distributions, data are presented as median (first quartile—Q1; third quartile—Q3). Statistical differences between groups were tested using parametric or nonparametric method, as appropriate. Two-tailed unpaired Student’s *t* test, or classical OLS regression model to adjust for potential confounders, was used as parametric methods, while Mann-Whitney *U* test, or quantile regression model with inference based on median to adjust for potential confounders, was used as nonparametric methods. For luciferase assay, comparison between groups was determined by one-way analysis of variance (ANOVA) and Bonferroni correction post hoc tests were carried out to detect significant differences between specific groups. Relationships between variables were assessed through an extension of Spearman’s rank correlations [53], which also allowed to adjusted correlations for age using probability-scale residuals. For all the statistical tests, *p* < 0.05 was considered statistically significant. R software environment for statistical computing, Version 3.6.0 (http://www.R-project.org), was used for all statistical analysis.

#### Experimental procedures

For each reported investigation, the experiments have been performed always by the same investigator. Same batch reagents and same instruments have been used. Technical and biological replicates were also considered in order to exclude technical errors and biological variance. Controls for each group of experiments have been also used.

## Supplementary information

**Additional file 1: Figure S1.** Correlation between VAT and PBL *ANKRD26* mRNA. **Table S1.** DNA methylation enrichment in the *ANKRD26* promoter. **Table S2.** Individual DNA methylation at the CpG sites, -689, -659, and -651 of the *ANKRD26* promoter in relation to *ANKRD26* mRNA expression and to anthropometric, metabolic and inflammatory parameters. **Table S3.** Reference gene’s threshold cycle (Ct) variability in lean and obese individuals.

## Data Availability

The data sets used and/or analysed during this study are available from the corresponding authors on reasonable request.

## Abbreviations

ANKRD26: Ankyrin Repeat Domain 26
ANOVA: One-way analysis of variance
BMI: Body mass index
CRP: C-reactive protein
CVDs: Cardiovascular diseases
HbA1c: Hemoglobin A1c
HDL-C: High-density lipoprotein cholesterol
HOMA-IR: Homeostatic model assessment of insulin resistance
IFNγ: Interferon γ
IL: Interleukin
IP-10: Interferon gamma-induced protein
LDL-C: Low-density lipoprotein cholesterol
MAF: Minor allele frequency
MCP1: Monocyte chemotactic protein 1
MeDIP-seq: Methylated DNA immuno-precipitation sequencing
MHO: Metabolically healthy obese
MIP1: Macrophage inflammatory protein 1
MUO: Metabolically unhealthy obese
PBL: Peripheral blood leukocytes
RANTES: Normal T cell expressed and secreted
SNPs: Single-nucleotide polymorphisms
T2D: Type 2 diabetes
TC: Total cholesterol
TG: Triglycerides
TNFα: Tumor necrosis factor α
VAT: Visceral adipose tissue
