# Supplementary material for: Epigenetic silencing of the ANKRD26 gene correlates to the pro-inflammatory profile and increased cardio-metabolic risk factors in human obesity
Source: Clin Epigenetics. 2019 Dec 4;11:181. doi: 10.1186/s13148-019-0768-0 (PMC6894277; doi:10.1186/s13148-019-0768-0)
Supplement: Supplementary file 1 — Additional file 1: Figure S1. Correlation between VAT and PBL ANKRD26 mRNA. Table S1. DNA methylation enrichment in the ANKRD26 promoter. Table S2. Individual DNA methylation at the CpG sites, -689, -659, and -651 of the ANKRD26 promoter in relation to ANKRD26 mRNA expression and to anthropometric, metabolic and inflammatory parameters. Table S3. Reference gene’s threshold cycle (Ct) variability in lean and obese individuals. [file 13148_2019_768_MOESM1_ESM.pdf]

Additional file 1

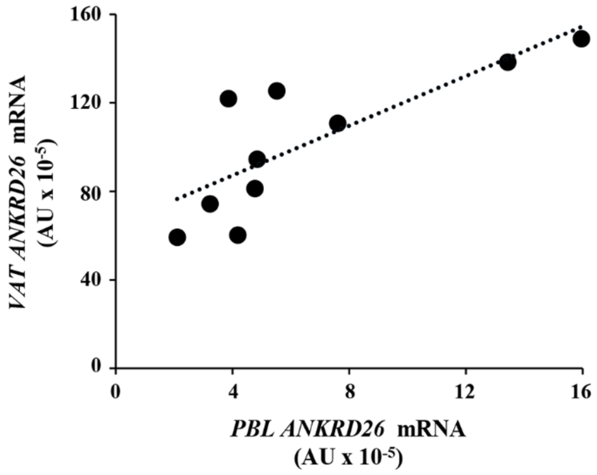

Fig S1. Correlation between VAT and PBL *ANKRD26* mRNA. Relationship between *ANKRD26* gene expression in VAT biopsies and PBL was assessed in lean (n=5) and obese (n=5) individuals by covariate-adjusted Spearman's rank-order correlation adjusted for age. r=0.850; p=0.014.

Additional file 1

Table S1. DNA methylation enrichment in the *ANKRD26* promoter. DNA methylation enrichment in the *ANKRD26* promoter. Percentage of DNA methylation of the 5 sub-regions analysed in lean (n=3) and obese subjects (n=3). S1: sub-region 1, S2: sub-region 2, S3: sub-region 3, S4: sub-region 4 and S5: sub-region 5. Values are mean ± SD. Statistical difference between the means of the two groups was assessed by two-tailed unpaired Student's t-test.

% CpG methylation			
	**Lean**	**Obese**	
**Position**	**mean ± SD**	**mean ± SD**	***p value***
S1, -991/-693	85.9 ± 2.1	85.2 ± 3.6	*n.s.*
S2, -716/-370	19.7 ± 8.2	26.7 ± 9.4	*<0.05*
S3, -349/-48	3.7 ± 4.6	1.0 ± 0.9	*n.s.*
S4, -68/+157	0.3 ± 0.5	0.5 ± 0.1	*n.s.*
S5, +134/+390	0.2 ± 0.1	0.4 ± 0.2	*n.s.*

Additional file 1

Table S2. Individual DNA methylation at the CpG sites, -689, -659, and -651 of the *ANKRD26* promoter in relation to *ANKRD26* mRNA expression and to anthropometric, metabolic and inflammatory parameters. Relationships were assessed in lean and obese individuals by covariate-adjusted Spearman's rank-order correlation adjusted for age. Number of individuals (*n*), correlation coefficient *r* and age-adjusted *p* value are shown on the table. TG: Triglyceride; TC: Total Cholesterol; HDL-C: High-Density Lipoprotein cholesterol; LDL-C: Low-Density Lipoprotein cholesterol; CRP: C-reactive protein; IL: Interleukin; IFNγ: Interferon γ; TNFα: Tumor necrosis factor α; IP-10: Interferon gamma-induced protein 10; MCP1: Monocyte chemotactic protein 1; MIP1: Macrophage inflammatory protein 1; RANTES: Regulated on activation, normal T cell expressed and secreted.

		% CpG methylation-689		***% CpG methylation-659***		***% CpG methylation-651***	
Parameters	***n***	**r**	***p value***	**r**	***p value***	**r**	***p value***
ANKRD26 mRNA	34	-0.689	*<0.001*	-0.458	*0.005*	-0.409	*0.029*
Age	34	0.243	*n.s.*	0.118	*n.s.*	0.268	*n.s.*
BMI	34	0.610	*<0.001*	0.700	*<0.001*	0.691	*<0.001*
Glucose	34	0.166	*n.s.*	0.114	*n.s.*	0.093	*n.s.*
TG	34	0.619	*<0.001*	0.538	*<0.001*	0.509	*0.002*
TC	34	0.139	*n.s.*	0.187	n.s.	0107	*n.s.*
HDL-C	34	-0.537	*0.010*	-0.523	*0.011*	-0.420	*0.020*
LDL-C	34	0.318	*n.s.*	0.464	*0.022*	0.315	*n.s.*
TG/HDL-C ratio	34	0.682	*<0.001*	0.604	*0.003*	0.547	*<0.001*
CRP	34	0.496	0.004	0.436	*0.011*	0.516	*0.001*
IL-1β	30	0.279	*n.s.*	0.227	*n.s.*	0.234	*n.s.*
IL-6	30	0.516	*0.013*	0.511	*0.003*	0.522	*0.002*
IL-7	30	0.160	*n.s.*	0.257	*n.s.*	0.142	*n.s.*
IL-9	30	0.123	*n.s.*	0.097	*n.s.*	-0.086	*n.s.*
IL-12	30	0.426	*0.018*	0.335	*n.s.*	0.460	*0.010*
IL-17	30	-0.015	*n.s.*	-0.062	*n.s.*	0.010	*n.s.*
IFN-γ	30	0.005	*n.s.*	0.154	*n.s.*	-0.149	*n.s.*
TNF-α	30	-0.015	*n.s.*	-0.020	*n.s.*	-0.134	*n.s.*
IL-8	30	0.669	*0.005*	0.639	*<0.001*	0.621	*0.002*
Eotaxin	30	0.018	*n.s.*	0.243	*n.s.*	-0.069	*n.s.*
IP-10	30	0.275	*n.s.*	0.359	*0.045*	0.281	*n.s.*
MCP-1	30	0.214	*n.s.*	0.443	*0.009*	0.248	*n.s.*
MIP-1α	30	0.388	*0.039*	0.289	*n.s.*	0.266	*n.s.*
MIP-1β	30	0.406	*n.s.*	0.327	*0.049*	0.343	*n.s.*
RANTES	30	0.511	*0.013*	0.495	*0.003*	0.463	*0.014*

Additional file 1

Table S3. Reference gene's threshold cycle (Ct) variability in lean and obese individuals. Variability of Ct for 4 different human reference genes, *β-ACTIN*, *ribosomal protein L13a* (*RPL13A*), *18S rRNA* and *28S rRNA*, was tested in PBL from lean and obese subjects in order to determine the appropriate reference gene for Quantitative Real-Time PCR assays. Values are mean ± SD. Statistical difference between the means of the two groups was assessed by two-tailed unpaired Student's t-test.

**Reference genes**	**Lean Ct (Mean ± SD)**	**Obese Ct (Mean ± SD)**	***p* value**
Β-ACTIN	13.15 ± 0.12	15.09 ± 0.94	0.052
RPL13A	19.13 ± 0.07	20.12 ± 0.56	0.384
18S	7.89 ± 0.26	11.60 ± 3.91	0.275
28S	9.27 ± 0.19	9.21 ± 0.34	0.709
